# 3,5-Dimethyl-1-(4-nitro­phen­yl)-1*H*-pyrazole

**DOI:** 10.1107/S1600536812009579

**Published:** 2012-03-10

**Authors:** Edward R. T. Tiekink, Solange M. S. V. Wardell, James L. Wardell

**Affiliations:** a Department of Chemistry, University of Malaya, 50603 Kuala Lumpur, Malaysia; b CHEMSOL, 1 Harcourt Road, Aberdeen AB15 5NY, Scotland; c Centro de Desenvolvimento Tecnológico em Saúde (CDTS), Fundação Oswaldo Cruz (FIOCRUZ), Casa Amarela, Campus de Manguinhos, Av. Brasil 4365, 21040-900 Rio de Janeiro, RJ, Brazil

## Abstract

In the title pyrazole derivative, C_11_H_11_N_3_O_2_, the benzene ring is twisted [dihedral angle = 31.38 (12)°] with respect to the pyrazole ring (r.m.s. deviation = 0.009 Å). The nitro group is effectively coplanar with the benzene ring to which it is attached [O—N—C—C torsion angle = −6.5 (3)°]. Supra­molecular chains along the *b* axis are formed owing to π–π inter­actions [3.8653 (2) Å] between translationally related mol­ecules involving both the five- and six-membered rings.

## Related literature

For the therapeutic importance of pyrazole compounds, see: Sil *et al.* (2005 ▶); Haddad *et al.* (2004 ▶). For the diverse pharmacological activities of pyrazole compounds, see: Bekhit *et al.* (2010 ▶, 2012) ▶; Higashi *et al.* (2006 ▶). For the synthesis, see: Butler & James (1982 ▶); Claramunt *et al.* (2006 ▶). For recently reported structures, see: Wardell *et al.* (2012 ▶); Baddeley *et al.* (2012 ▶).
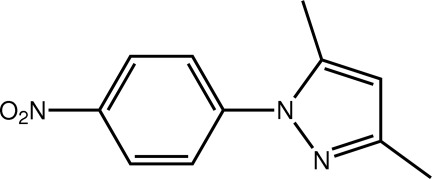


## Experimental

### 

#### Crystal data

C_11_H_11_N_3_O_2_
*M* *_r_* = 217.23
Orthorhombic, 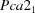

*a* = 21.3909 (13) Å
*b* = 3.8653 (2) Å
*c* = 12.4514 (8) Å
*V* = 1029.51 (11) Å^3^
*Z* = 4
Mo *K*α radiation
μ = 0.10 mm^−1^
*T* = 120 K
0.26 × 0.19 × 0.04 mm

#### Data collection

Rigaku Saturn724+ diffractometer
Absorption correction: multi-scan (*SADABS*; Sheldrick, 2007 ▶) *T*_min_ = 0.598, *T*_max_ = 1.000
6055 measured reflections
1202 independent reflections
1148 reflections with *I* > 2σ(*I*)
*R*_int_ = 0.046

#### Refinement

*R*[*F* ^2^ > 2σ(*F* ^2^)] = 0.037
*wR*(*F* ^2^) = 0.101
*S* = 1.09
1202 reflections
147 parameters
1 restraint
H-atom parameters constrained
Δρ_max_ = 0.16 e Å^−3^
Δρ_min_ = −0.20 e Å^−3^

### 

Data collection: *COLLECT* (Hooft, 1998 ▶); cell refinement: *DENZO* (Otwinowski & Minor, 1997 ▶) and *COLLECT*; data reduction: *DENZO* and *COLLECT*; program(s) used to solve structure: *SHELXS97* (Sheldrick, 2008 ▶); program(s) used to refine structure: *SHELXL97* (Sheldrick, 2008 ▶); molecular graphics: *ORTEP-3* (Farrugia, 1997 ▶) and *DIAMOND* (Brandenburg, 2006 ▶); software used to prepare material for publication: *publCIF* (Westrip, 2010 ▶).

## Supplementary Material

Crystal structure: contains datablock(s) global, I. DOI: 10.1107/S1600536812009579/hg5187sup1.cif

Structure factors: contains datablock(s) I. DOI: 10.1107/S1600536812009579/hg5187Isup2.hkl

Supplementary material file. DOI: 10.1107/S1600536812009579/hg5187Isup3.cml

Additional supplementary materials:  crystallographic information; 3D view; checkCIF report

## Figures and Tables

**Fig. 1.**
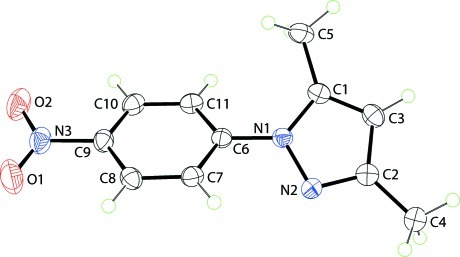
The molecular structure of (I) showing the atom-labelling scheme and displacement ellipsoids at the 50% probability level.

**Fig. 2.**
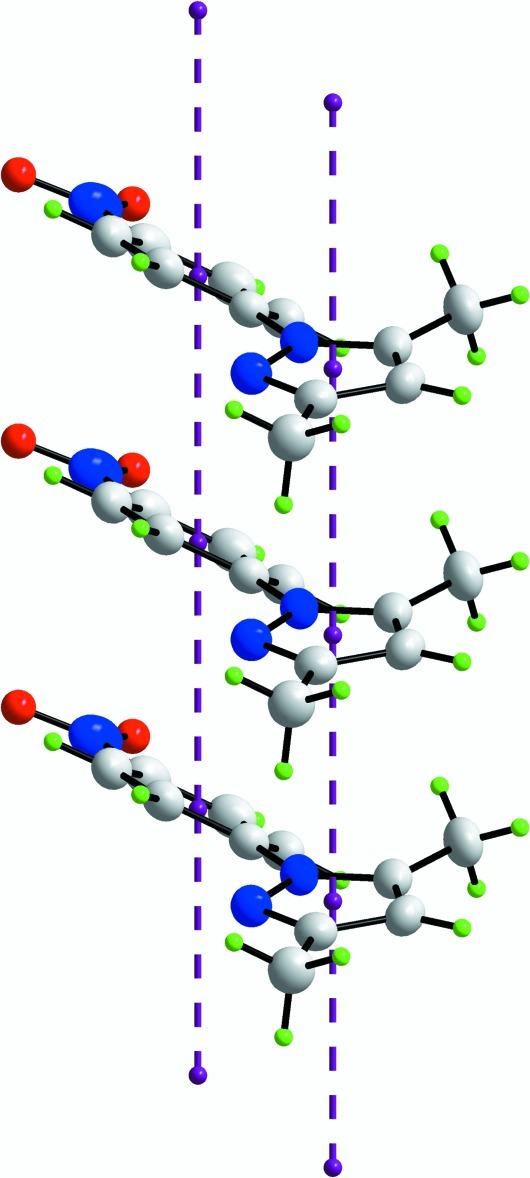
A view of the linear supramolecular chain in (I) sustained by π–π interactions (purple dashed lines) along the b axis.

**Fig. 3.**
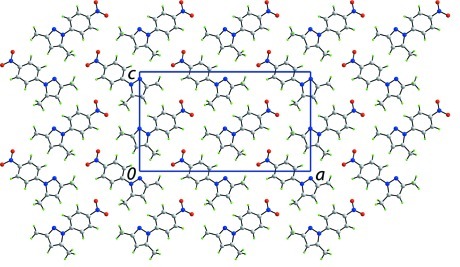
A view in projection down the b axis of the packing of supramolecular chains in (I).

## supplementary crystallographic information

### Comment

Pyrazoles are key structures in numerous compounds of therapeutic importance (Sil *et al.*, 2005, Haddad *et al.*, 2004). Compounds containing this ring system are known to display diverse pharmacological activities, for example as anti-malarial agents (Bekhit *et al.*, 2012), anti-inflammatory agents (Bekhit *et al.*, 2010), and against cardiovascular disease (Higashi *et al.*, 2006). A general route to pyrazole derivatives involves reaction of an arylhydrazine, ArNHNH_2_, with a β-dicarbonyl compound, *R*'COCH_2_COY. In connection with recent structural studies (Wardell *et al.*, 2012; Baddeley *et al.*, 2012), we now wish to report the structure of the title compound, (I), prepared from 4-O_2_NC_6_H_4_NHNH_2_ and MeCOCH_2_COMe.

In (I), Fig. 1, the pyrazole ring is planar with a r.m.s. deviation for the fitted atoms of 0.009 Å. The benzene ring is twisted out of this plane forming a dihedral angle of 31.38 (12)°. The nitro group is effectively co-planar with the benzene ring to which it is connected as seen in the value of the O1—N3—C9—C8 torsion angle of -6.5 (3)°.

The most prominent intermolecular interactions in the crystal structure of (I) are of the type π–π. These form between translationally related molecules along the *b* axis, involving both the five- and six-membered rings, and therefore, the ring centroid separations are 3.8653 (2) Å, Fig. 2. Columns pack with no specific intermolecular interactions between them, Fig. 3.

### Experimental

A solution of 4-O_2_NC_6_H_4_NHNH_2_ (2 mmol) and MeCOCH_2_COMe (2 mmol) in EtOH (20 ml) was refluxed for 1 h. The solution was maintained at room temperature and crystals were collected after a few days, *M*.pt: 373–375 K; lit. *M*.pt: 373–375 K (Butler & James, 1982). NMR spectra were identical with those reported (Claramunt *et al.*, 2006). IR ν: 3300, 1608, 1597, 1570, 1518, 1504, 1414, 1334, 1301, 1273, 1176, 1110, 1934, 982, 854, 825, 801, 749, 689, 640, 502 cm^-1^.

### Refinement

The C-bound H atoms were geometrically placed (C—H = 0.95–0.98 Å) and refined as riding with *U*_iso_(H) = 1.2–1.5*U*_eq_(C). In the absence of significant anomalous scattering effects, 515 Friedel pairs were averaged in the final refinement.

### Crystal data

**Table:**

C_11_H_11_N_3_O_2_	*F*(000) = 456
*M**_r_* = 217.23	*D*_x_ = 1.402 Mg m^−^^3^
Orthorhombic, *P**c**a*2_1_	Mo *K*α radiation, λ = 0.71073 Å
Hall symbol: P 2c -2ac	Cell parameters from 6528 reflections
*a* = 21.3909 (13) Å	θ = 2.9–27.5°
*b* = 3.8653 (2) Å	µ = 0.10 mm^−^^1^
*c* = 12.4514 (8) Å	*T* = 120 K
*V* = 1029.51 (11) Å^3^	Plate, light-yellow
*Z* = 4	0.26 × 0.19 × 0.04 mm

### Data collection

**Table:**

Rigaku Saturn724+ diffractometer	1202 independent reflections
Radiation source: Rotating Anode	1148 reflections with *I* > 2σ(*I*)
Confocal monochromator	*R*_int_ = 0.046
Detector resolution: 28.5714 pixels mm^-1^	θ_max_ = 27.5°, θ_min_ = 3.3°
profile data from ω–scans	*h* = −27→25
Absorption correction: multi-scan (*SADABS*; Sheldrick, 2007)	*k* = −4→5
*T*_min_ = 0.598, *T*_max_ = 1.000	*l* = −16→10
6055 measured reflections	

### Refinement

**Table:**

Refinement on *F*^2^	Primary atom site location: structure-invariant direct methods
Least-squares matrix: full	Secondary atom site location: difference Fourier map
*R*[*F*^2^ > 2σ(*F*^2^)] = 0.037	Hydrogen site location: inferred from neighbouring sites
*wR*(*F*^2^) = 0.101	H-atom parameters constrained
*S* = 1.09	*w* = 1/[σ^2^(*F*_o_^2^) + (0.0563*P*)^2^ + 0.2711*P*] where *P* = (*F*_o_^2^ + 2*F*_c_^2^)/3
1202 reflections	(Δ/σ)_max_ < 0.001
147 parameters	Δρ_max_ = 0.16 e Å^−^^3^
1 restraint	Δρ_min_ = −0.20 e Å^−^^3^

### Special details

**Table:**

Geometry. All s.u.'s (except the s.u. in the dihedral angle between two l.s. planes) are estimated using the full covariance matrix. The cell s.u.'s are taken into account individually in the estimation of s.u.'s in distances, angles and torsion angles; correlations between s.u.'s in cell parameters are only used when they are defined by crystal symmetry. An approximate (isotropic) treatment of cell s.u.'s is used for estimating s.u.'s involving l.s. planes.
Refinement. Refinement of *F*^2^ against ALL reflections. The weighted *R*-factor *wR* and goodness of fit *S* are based on *F*^2^, conventional *R*-factors *R* are based on *F*, with *F* set to zero for negative *F*^2^. The threshold expression of *F*^2^ > 2σ(*F*^2^) is used only for calculating *R*-factors(gt) *etc*. and is not relevant to the choice of reflections for refinement. *R*-factors based on *F*^2^ are statistically about twice as large as those based on *F*, and *R*- factors based on ALL data will be even larger.

### Fractional atomic coordinates and isotropic or equivalent isotropic displacement parameters (Å^2^)

**Table:**

	*x*	*y*	*z*	*U*_iso_*/*U*_eq_	
O1	0.25661 (11)	0.8154 (7)	0.1996 (2)	0.0529 (6)	
O2	0.19518 (9)	0.7062 (6)	0.0666 (2)	0.0479 (6)	
N1	0.44986 (9)	0.1859 (5)	−0.12778 (15)	0.0229 (4)	
N2	0.50113 (9)	0.0689 (5)	−0.07183 (17)	0.0246 (4)	
N3	0.24680 (11)	0.7002 (5)	0.1097 (2)	0.0348 (5)	
C1	0.45879 (11)	0.1711 (6)	−0.23678 (19)	0.0246 (5)	
C2	0.54135 (11)	−0.0271 (6)	−0.14708 (19)	0.0260 (5)	
C3	0.51697 (11)	0.0328 (6)	−0.2510 (2)	0.0274 (5)	
H3	0.5371	−0.0138	−0.3175	0.033*	
C4	0.60413 (11)	−0.1723 (7)	−0.1187 (2)	0.0308 (5)	
H4A	0.6188	−0.0674	−0.0517	0.046*	
H4B	0.6339	−0.1211	−0.1765	0.046*	
H4C	0.6008	−0.4234	−0.1095	0.046*	
C5	0.41435 (12)	0.3090 (7)	−0.3190 (2)	0.0313 (5)	
H5A	0.3823	0.1346	−0.3344	0.047*	
H5B	0.4372	0.3637	−0.3850	0.047*	
H5C	0.3943	0.5191	−0.2914	0.047*	
C6	0.39825 (10)	0.3116 (6)	−0.06913 (18)	0.0225 (5)	
C7	0.40877 (11)	0.4650 (6)	0.03071 (18)	0.0251 (5)	
H7	0.4502	0.4872	0.0574	0.030*	
C8	0.35886 (11)	0.5849 (6)	0.0909 (2)	0.0267 (5)	
H8	0.3654	0.6858	0.1595	0.032*	
C9	0.29891 (11)	0.5547 (6)	0.0488 (2)	0.0274 (5)	
C10	0.28776 (11)	0.4044 (6)	−0.0498 (2)	0.0282 (5)	
H10	0.2463	0.3888	−0.0769	0.034*	
C11	0.33738 (11)	0.2765 (6)	−0.1088 (2)	0.0259 (5)	
H11	0.3302	0.1657	−0.1758	0.031*	

### Atomic displacement parameters (Å^2^)

**Table:**

	*U*^11^	*U*^22^	*U*^33^	*U*^12^	*U*^13^	*U*^23^
O1	0.0478 (12)	0.0660 (13)	0.0448 (13)	0.0087 (12)	0.0146 (10)	−0.0132 (11)
O2	0.0249 (9)	0.0550 (13)	0.0640 (14)	0.0063 (9)	0.0092 (9)	0.0016 (11)
N1	0.0223 (9)	0.0249 (9)	0.0216 (9)	−0.0003 (7)	−0.0012 (7)	0.0011 (7)
N2	0.0227 (8)	0.0262 (10)	0.0249 (9)	0.0020 (7)	−0.0012 (7)	0.0005 (9)
N3	0.0300 (11)	0.0318 (11)	0.0427 (13)	0.0032 (9)	0.0117 (10)	0.0062 (10)
C1	0.0273 (11)	0.0239 (10)	0.0225 (10)	−0.0041 (9)	−0.0019 (9)	−0.0004 (9)
C2	0.0279 (11)	0.0224 (10)	0.0275 (11)	−0.0021 (9)	−0.0009 (9)	−0.0003 (9)
C3	0.0330 (12)	0.0252 (11)	0.0241 (11)	−0.0025 (9)	0.0028 (9)	−0.0035 (9)
C4	0.0271 (11)	0.0329 (13)	0.0324 (13)	0.0019 (9)	0.0016 (9)	−0.0002 (11)
C5	0.0337 (12)	0.0368 (14)	0.0233 (10)	−0.0035 (11)	−0.0046 (9)	0.0038 (10)
C6	0.0236 (10)	0.0202 (10)	0.0238 (11)	0.0000 (7)	0.0009 (8)	0.0023 (9)
C7	0.0245 (10)	0.0252 (11)	0.0254 (10)	0.0001 (8)	−0.0016 (9)	0.0033 (10)
C8	0.0296 (11)	0.0260 (11)	0.0246 (11)	−0.0015 (9)	0.0037 (9)	0.0019 (9)
C9	0.0250 (11)	0.0260 (11)	0.0312 (12)	0.0019 (9)	0.0073 (9)	0.0059 (10)
C10	0.0206 (10)	0.0297 (11)	0.0345 (13)	−0.0020 (9)	−0.0014 (9)	0.0060 (10)
C11	0.0270 (10)	0.0240 (11)	0.0268 (11)	−0.0025 (9)	−0.0042 (9)	0.0012 (9)

### Geometric parameters (Å, º)

**Table:**

O1—N3	1.222 (3)	C4—H4C	0.9800
O2—N3	1.228 (3)	C5—H5A	0.9800
N1—C1	1.372 (3)	C5—H5B	0.9800
N1—N2	1.376 (3)	C5—H5C	0.9800
N1—C6	1.410 (3)	C6—C7	1.396 (3)
N2—C2	1.325 (3)	C6—C11	1.399 (3)
N3—C9	1.461 (3)	C7—C8	1.384 (3)
C1—C3	1.366 (3)	C7—H7	0.9500
C1—C5	1.495 (3)	C8—C9	1.390 (3)
C2—C3	1.414 (3)	C8—H8	0.9500
C2—C4	1.498 (3)	C9—C10	1.379 (4)
C3—H3	0.9500	C10—C11	1.382 (3)
C4—H4A	0.9800	C10—H10	0.9500
C4—H4B	0.9800	C11—H11	0.9500
			
C1—N1—N2	112.11 (19)	C1—C5—H5B	109.5
C1—N1—C6	129.48 (19)	H5A—C5—H5B	109.5
N2—N1—C6	118.37 (19)	C1—C5—H5C	109.5
C2—N2—N1	104.56 (19)	H5A—C5—H5C	109.5
O1—N3—O2	123.2 (2)	H5B—C5—H5C	109.5
O1—N3—C9	119.0 (2)	C7—C6—C11	120.4 (2)
O2—N3—C9	117.8 (2)	C7—C6—N1	118.8 (2)
C3—C1—N1	105.7 (2)	C11—C6—N1	120.8 (2)
C3—C1—C5	129.1 (2)	C8—C7—C6	120.0 (2)
N1—C1—C5	125.0 (2)	C8—C7—H7	120.0
N2—C2—C3	111.2 (2)	C6—C7—H7	120.0
N2—C2—C4	121.4 (2)	C7—C8—C9	118.6 (2)
C3—C2—C4	127.4 (2)	C7—C8—H8	120.7
C1—C3—C2	106.4 (2)	C9—C8—H8	120.7
C1—C3—H3	126.8	C10—C9—C8	122.1 (2)
C2—C3—H3	126.8	C10—C9—N3	119.5 (2)
C2—C4—H4A	109.5	C8—C9—N3	118.4 (2)
C2—C4—H4B	109.5	C9—C10—C11	119.4 (2)
H4A—C4—H4B	109.5	C9—C10—H10	120.3
C2—C4—H4C	109.5	C11—C10—H10	120.3
H4A—C4—H4C	109.5	C10—C11—C6	119.5 (2)
H4B—C4—H4C	109.5	C10—C11—H11	120.3
C1—C5—H5A	109.5	C6—C11—H11	120.3
			
C1—N1—N2—C2	−1.6 (2)	N2—N1—C6—C11	−148.7 (2)
C6—N1—N2—C2	−179.38 (19)	C11—C6—C7—C8	−0.3 (3)
N2—N1—C1—C3	1.4 (3)	N1—C6—C7—C8	−178.8 (2)
C6—N1—C1—C3	178.9 (2)	C6—C7—C8—C9	−1.2 (3)
N2—N1—C1—C5	−174.1 (2)	C7—C8—C9—C10	1.1 (4)
C6—N1—C1—C5	3.4 (4)	C7—C8—C9—N3	−176.4 (2)
N1—N2—C2—C3	1.1 (2)	O1—N3—C9—C10	176.0 (2)
N1—N2—C2—C4	−179.9 (2)	O2—N3—C9—C10	−5.4 (3)
N1—C1—C3—C2	−0.7 (3)	O1—N3—C9—C8	−6.5 (3)
C5—C1—C3—C2	174.6 (2)	O2—N3—C9—C8	172.1 (2)
N2—C2—C3—C1	−0.3 (3)	C8—C9—C10—C11	0.5 (4)
C4—C2—C3—C1	−179.2 (2)	N3—C9—C10—C11	178.0 (2)
C1—N1—C6—C7	−147.5 (2)	C9—C10—C11—C6	−2.0 (3)
N2—N1—C6—C7	29.9 (3)	C7—C6—C11—C10	1.9 (3)
C1—N1—C6—C11	34.0 (3)	N1—C6—C11—C10	−179.6 (2)
